# News and Coming Events

**Published:** 1909-05-08

**Authors:** 


### NEWS AND COMING EVENTS.

THE Emperor of Austria has conferred upon Dr. Carl Harrer, Physician to the Austro-Hungarian Embassy and Consulate-General in London, the Knighthood of the Distinguished Order of the Iron Crown of Austria.

THE annual dinner of the Royal Sanitary Institute will be held on May 11 at the Langham Hotel, when the chair will be taken by the Duke of Northumberland, the President of the Institute.

DR. J. LUCAS-CHAMPIONNIÈRE, President of the International Society of Surgery, will deliver the annual address to the Cardiff Medical Society on Friday, June 4. The address will deal with the Modern Treatment of Fractures.

THE fifth International Congress of Dental Surgery will be held at Berlin from August 23 to 28. The work of the Congress will be distributed among twelve sections. The General Secretary is Dr. Schaeffer-Stuckert, of Frankfort-on-Main (Kettenhofweg, 29), to whom all communications relative to the Congress should be addressed. In connection with the Congress there will be an exhibition.

A FESTIVAL banquet in aid of the maintenance fund of the Mount Vernon Hospital for Consumption and Diseases of the Chest, Hampstead and Northwood, will be held at the Hôtel Cecil on Thursday, May 13, at 7.30 P.M., when Viscount Clifden, Deputy-Chairman of the Committee of Management, will preside.

HIS Excellency the Ambassador of the United States of America (Mr. Whitelaw Reid) has consented to preside at a Festival Dinner to be held at the Hôtel Cecil on Wednesday, June 9, in the hope that he may be instrumental in bringing substantial help to the work of the London Fever Hospital, Liverpool Road, N. Invitation cards will be forwarded to those who wish to be present by Major W. Christie, the Secretary of the Hospital, who will also receive and acknowledge contributions.

THE annual meeting of the Invalid Children's Aid Association will be held by kind permission of Lord and Lady Newlands at 16 Grosvenor Place, S.W., on Tuesday, May 25, at 3 P.M. Field-Marshal Lord Grenfell will preside, and the Bishop of Stepney and Sir Alfred Fripp, F.R.C.S., will be among the speakers. Tickets of admission can be obtained from the Secretary, 69 Denison House, Vauxhall Bridge Road, S.W.

THE Annual Middlesex Hospital Founder's Day Commemoration Service will be held in the Hospital Chapel on Wednesday, May 19, at 3.30 P.M., when a sermon will be preached by the Rev. Herbert E. Gunson, M.A., the Chaplain. After the service visitors will have the opportunity of inspecting the wards of the hospital, and will be entertained at tea by the invitation of Lord Cheylesmore and members of the Weekly Board of Governors, in whose name the cards of invitation are issued.

BY the kind permission of Mary Countess of Ilchester, a concert in aid of the Chelsea Hospital for Women will be held in the Garden Ballroom at Holland House, Kensington, on Friday, June 11, at 3 P.M., when the Gardens at Holland House will be thrown open to the audience. The hospital is now in need of financial assistance. An amount of £2,000 beyond its regular income is needed annually to provide for ordinary expenditure. The concert will be under Royal patronage.

A SPECIAL meeting of the Irish Medical Schools and Graduates' Association will be held at Harrogate on Saturday, May 22. Arrangements have been made with the Majestic Hotel to permit members to remain until Monday and visit places of interest in the neighbourhood. Full particulars may be obtained from the hon. secretary, 30 Myddelton Square, London, E.C.

A SPECIAL meeting of the Dermatological Section of the Royal Society of Medicine will be held at 20 Hanover Square, London, W., on Thursday, May 20, at 5 P.M., when Dr. Louis Wickham, of the Radium Institute, Paris, will give a lecture-demonstration on "The Therapeutics of Radium."

THE annual general meeting of the Medical Defence Union (Limited by Guarantee), will be held at the medical library of University College, Bristol, by permission of the authorities, on Thursday, May 27, at 4.30 P.M., when the Annual Report will be presented and the usual statutory business transacted.

THE tenth meeting of the Departmental Committee appointed by the Lord President of the Council to consider the working of the Midwives Act was held at the Privy Council Office on April 28, Mr. Almeric W. FitzRoy, the Clerk of the Council, presiding. Five witnesses—personally connected either with obstetrical nursing or with its organisation—attended and gave evidence.

THE Ingleby Lecture for 1909 in the University of Birmingham will be delivered on Thursday, May 27, at 4 P.M., in the Medical Lecture Theatre, by Sir Thomas Barlow, Bt., K.C.V.O., M.D., F.R.C.P., Professor of Clinical Medicine, University College Hospital, London. The subject will be "Raynaud's Disease and Erythromelalgia: a Summary and a Review."

THE fourth International Milk Trade Congress will be held, under the patronage of His Royal Highness the Grand Duke Joseph, at Buda-Pesth, from the 6th to the 11th of June, this year. The questions to be discussed are divided into three groups—legislative and administrative, hygienic and veterinary, and industrial. It is announced that some 600 persons will take part in the Congress, and that sixty communications have already been received by the secretary to the Congress.

A SESSIONAL meeting of the Royal Sanitary Institute will be held in the Parkes Museum, Margaret Street, W., on Wednesday, May 12, at 8 P.M., when a discussion on "The Passage of Excreta through House Drains" will be opened by Mr. H. A. Roechling, M.Inst.C.E., F.G.S., Fellow of the Institute. The chair will be taken at 8 P.M. by Mr. H. D. Searles Wood, F.R.I.B.A., Chairman of the Council. Tickets for admission of visitors may be had on application from Mr. E. White Wallis, Secretary. Tea and coffee will be served after the meeting.

THE Faculty of Medicine of the University of Birmingham announces that the remaining two lectures of the course upon "The Essentials of Physiological History" to be delivered by Dr. David Fraser Harris, M.D.Glas., B.Sc.Lond., F.R.S.E. (Lecturer in Physiology), during the present Summer Session in the Medical Theatre, will take place at 4 P.M. upon the following dates: May 13—"The History of Knowledge of the Respiration"; May 20—"The History of Knowledge of the Nervous System." Members of the medical profession and students of medicine are invited to attend.

